# Transcript availability dictates the balance between strand-asynchronous and strand-coupled mitochondrial DNA replication

**DOI:** 10.1093/nar/gky852

**Published:** 2018-09-20

**Authors:** Tricia J Cluett, Gokhan Akman, Aurelio Reyes, Lawrence Kazak, Alice Mitchell, Stuart R Wood, Antonella Spinazzola, Johannes N Spelbrink, Ian J Holt

**Affiliations:** 1 MRC Mitochondrial Biology Unit, University of Cambridge, Cambridge CB1 9SY, UK; 2 MRC Mill Hill Laboratory, Mill Hill, London, UK; 3 Department of Clinical Movement Neurosciences, Institute of Neurology, Royal Free Campus, University College London, London NW3 2PF, UK; 4 MRC Centre for Neuromuscular Diseases, UCL Institute of Neurology and National Hospital for Neurology and Neurosurgery, London, UK.; 5 Department of Pediatrics, Radboud Centre for Mitochondrial Medicine, Radboud University Medical Centre, Geert Grooteplein 10, 6500 HB, Nijmegen, The Netherlands; 6 Biodonostia Health Research Institute, 20014 San Sebastián, Spain and IKERBASQUE, Basque Foundation for Science, 48013 Bilbao, Spain

## Abstract

Mammalian mitochondria operate multiple mechanisms of DNA replication. In many cells and tissues a strand-asynchronous mechanism predominates over coupled leading and lagging-strand DNA synthesis. However, little is known of the factors that control or influence the different mechanisms of replication, and the idea that strand-asynchronous replication entails transient incorporation of transcripts (aka bootlaces) is controversial. A firm prediction of the bootlace model is that it depends on mitochondrial transcripts. Here, we show that elevated expression of Twinkle DNA helicase in human mitochondria induces bidirectional, coupled leading and lagging-strand DNA synthesis, at the expense of strand-asynchronous replication; and this switch is accompanied by decreases in the steady-state level of some mitochondrial transcripts. However, in the so-called minor arc of mitochondrial DNA where transcript levels remain high, the strand-asynchronous replication mechanism is instated. Hence, replication switches to a strand-coupled mechanism only where transcripts are scarce, thereby establishing a direct correlation between transcript availability and the mechanism of replication. Thus, these findings support a critical role of mitochondrial transcripts in the strand-asynchronous mechanism of mitochondrial DNA replication; and, as a corollary, mitochondrial RNA availability and RNA/DNA hybrid formation offer means of regulating the mechanisms of DNA replication in the organelle.

## INTRODUCTION

The maintenance and expression of mitochondrial DNA (mtDNA) is essential for life, as its products are required for aerobic energy production. One of the proteins critical for mtDNA maintenance is Twinkle DNA helicase (1), and mutations in *TWINKLE* (*PEO1*) are a well-established cause of human disease (2). Twinkle is a hexameric ring helicase analogous to MCM2-7 of nuclear DNA replication (3,4), which was identified on the basis of its similarity to the gp4 primase-helicase of the T7 phage (2). However, mammalian Twinkle does not display detectable primase activity (5), and therefore appears not to be able to synthesize the short RNAs (4–16 nts) that support classical coordinated DNA replication. Twinkle is the presumed replicative DNA helicase of animal mitochondria, and its elevated expression *in vivo* increases mtDNA copy number and decreases transcript levels (6), although its effect on the mechanism of replication was not assessed.

**Figure 1.**
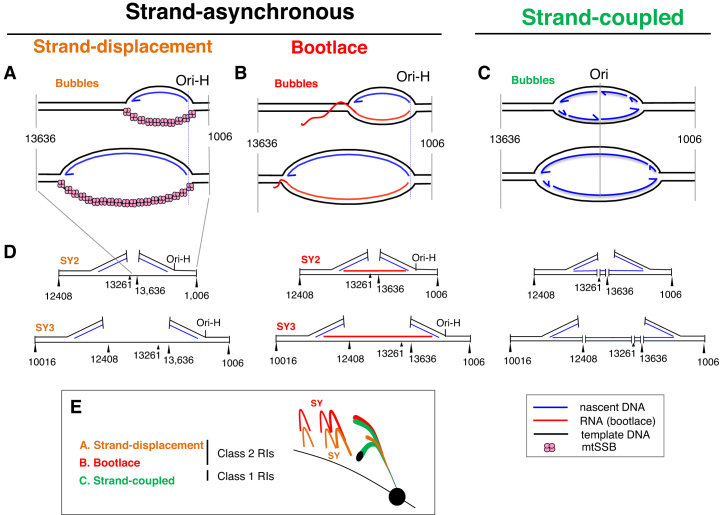
Models of mammalian mtDNA replication. Two alternative mechanisms of strand-asynchronous replication have been proposed: (**A**) the strand-displacement model that depends on protein (mtSSB) to coat the displaced lagging-strand template, and (**B**) the bootlace mechanism that relies on mature transcripts. (**C**) Intermediates with the properties of conventional (concurrent) leading and lagging-strand DNA synthesis have also been reported in mammalian mitochondria suggesting that they also operate a strand-coupled (SC) mechanism of replication. (**D**) Restriction digestion of the intermediates of strand-asynchronous replication will not cut at sites that are single-stranded DNA (in the case of many restriction enzymes) or where there is RNA/DNA hybrid, which produces atypical structures compared to SC replication as illustrated, and these form so-called supra-Y (SY) arcs when resolved by 2D-AGE (**E**).

Mitochondrial DNA operates multiple mechanisms of replication (7,8); in mammals a strand-asynchronous mechanism predominates in most cells and tissues. It was suggested that single-stranded DNA binding protein (mtSSB) coated the displaced lagging-strand template during the prolonged interval between the initiation of first and second strand DNA synthesis (Figure 1A) (9). Later, the discovery and characterization of RNA/DNA hybrids associated with replicating mtDNA led to the proposal that leading strand DNA synthesis is accompanied by hybridization of mitochondrial transcripts to the lagging-strand template (Figure 1B) ((10) and references therein). Additionally, mitochondrial replication intermediates (mtRIs) with the properties of the products of coupled leading and lagging-strand DNA synthesis are found in mammalian and avian mitochondria (Figure 1C) (11–14). Since its first crude conception (15), the ‘bootlace’ mechanism of mtDNA replication has struggled to gain widespread acceptance, despite inter-strand cross-linking experiments having shown that the RNA/DNA hybrids form on most if not all replicating mtDNA molecules in intact organelles and cells (10), and the evident risk that would accompany the uniquely large fragile region envisaged by the alternative ‘strand-displacement’ model (16). A more recent analysis of the mtDNA binding pattern of mtSSB in HeLa cells was consistent with some replicating molecules having extensive single-stranded regions (17); however, the extent of loss of RNA/DNA hybrids during the mitochondrial isolation step was not assessed, but likely to have been considerable based on the relatively poor quality of mtRIs from cultured cells compared to solid tissues in other studies (e.g. (10)). Given the continuing controversy, it is important to manipulate the replication process in ways that test the predictions of the different models. The most straightforward prediction of the bootlace model of mtDNA replication is that it depends on mitochondrial transcripts, whereas coating of the displaced lagging strand with mtSSB during replication would be transcript-independent. Here, we show that strand-asynchronous mtDNA replication is in abeyance when and where transcripts are scarce, and that it is substituted by the coupled leading and lagging strand mtDNA replication mechanism.

## MATERIALS AND METHODS

### Generation of constructs and cell lines carrying TWINKLE transgenes

A cDNA of TWINKLE was cloned into vector pcDNA5/FRT/TO (Invitrogen). In addition to the wild-type untagged form of the gene, Stratagene's Site-directed Mutagenesis kit was used to create point mutants G575D (mut1), D311A (mut2) and E266A (mut3) (Supplementary Figure S1), and to add a hemagglutinin (Twk-HA), FLAG-Strep (Twk-F), or a myc-his (Twk-myc) tag at the carboxyl (C)-terminus, essentially as previously (18). All three mutants were expected to have reduced helicase activity (5,18,19). HEK293 Flp-In™ T-REx™ (hereafter HEK293T) cells (Invitrogen) were transfected with 0.2 μg of pcDNA5.Twinkle and 1 μg of poG44, using lipofectamine2000 (Invitrogen) according to the manufacturer's instructions. HEK293T cells were cultured in Dulbecco's Modified Eagle's Medium (DMEM), supplemented with 10% tetracycline-free fetal bovine serum, 15 μg/ml blasticidin and 100 μg/ml zeocin, prior to transfection with pcDNA5 plasmids. After transfection the medium contained 100 μg/ml hygromycin and no zeocin. The transgenes were induced by the addition of doxycycline for the times and with the doses indicated in the figures.

### Nucleic acid isolation, digestion and modification

Total DNA was extracted from HEK293T cells by lysis, protease digestion and successive phenol and chloroform extractions, as previously (20). Restriction digestions (New England Biolabs) were performed on 10 μg aliquots of whole cell nucleic acid. RNA/DNA hybrid was digested by incubation of isolated nucleic acids with 1 U of *Escherichia coli* Ribonuclease HI (Promega). Single-stranded nucleic acid was digested with 1 U of S1 nuclease (Promega) for 90 s at 37°C.

### Neutral two-dimensional agarose gel electrophoresis and hybridization

Neutral two-dimensional agarose gel electrophoresis (2D-AGE) for fragments of 3–6 kb was according to the standard method (21,22). Fragments larger than 5 kb were separated in the first dimension at 0.7–0.9 V/cm for 20–24 h on slab gels of between 0.28 and 0.35% (w/v) agarose, at room temperature. Second dimension electrophoresis was for 27–72 h at 36–100 V in gels of 0.58–0.875% agarose, at 4 or 25°C.

In-gel digestion for fork-direction gels was carried out as described previously (11,22). Briefly, 1D gel slices were washed twice with 10 mM Tris, 0.1 mM EDTA pH 8.0 for 30 min at room temperature. After equilibrating the gel slice with 1× restriction enzyme buffer (New England Biolabs) for 1 h at room temperature, 100 U of restriction enzyme was added directly to the surface of the gel, after incubation at 37°C for 3 h; a further 50 U of enzyme were added and the incubation continued overnight. After incubation, the gel slice was washed first with TE pH 8.0 for 30 min at room temperature and then with TBE 1× for 15 min at room temperature. Thereafter, the standard 2D-AGE procedure was followed.

After electrophoresis, the DNA was depurinated with 250 mM HCl for 20 min, denatured in 0.5 M NaOH, 1.5 M NaCl for 30 min, and neutralized in 0.5 M Tris–HCl (pH 7.4), 1.5 M NaCl for 30 min. After transfer to nylon membranes (Magnaprobe, Osmonics Inc.) and UV cross-linking of the nucleic acids to the membrane by exposure to 120 mJ/cm^2^, 256 nM light, the membranes were hybridized to denatured, radiolabeled, DNA probes. Radiolabeled DNA probes were generated from fragments of human mtDNA PCR-amplified using pairs of oligonucleotide primers (Sigma) (listed in supplementary information), based on the reference sequence (23). Labeling used 50 μCi of [α-^32^P] dCTP (3000 Ci/mmol, Perkin Elmer), 50 ng of heat denatured DNA and DNA labeling beads (GE Healthcare), incubated for 30 min at 37°C.

### Fractionation of nascent strands

For the detection of nascent strands, restriction digested DNA samples were denatured in formamide and fractionated by 1D-AGE on a 2.35–2.5% Nu-Sieve 3:1 agarose (Lonza) gels in 10 mM sodium borate buffer, as previously described (24,25). After transfer to solid support, nucleic acids were UV cross-linked to the membrane and hybridized with radiolabeled strand-specific RNA probes, using T7-maxiscript kit (Ambion) as per the manufacturer's instructions. H-strand riboprobe templates were generated via PCR, using the primer pairs listed in the supplementary section.

For both 1D and 2D blots, membranes were hybridized to radiolabeled probes by overnight incubation at 60–65°C in 7% SDS, 0.25 M sodium phosphate pH 7.2. Post-hybridization washes were 1× SSC twice, 1× SSC, 0.1% SDS twice, each for 20 min at 60–65°C. Filters were exposed to X-ray film or phosphorscreens for 1–10 days.

### Quantitative PCR

Q-PCR for mtDNA copy number and Q-RT-PCR for mRNA quantification were performed as described previously (26) with the primers, probes and conditions listed in supplementary information.

## RESULTS

### Elevated expression of wild-type Twinkle induces a switch from strand-asynchronous to strand-coupled mtDNA replication in human cells

**Figure 2.**
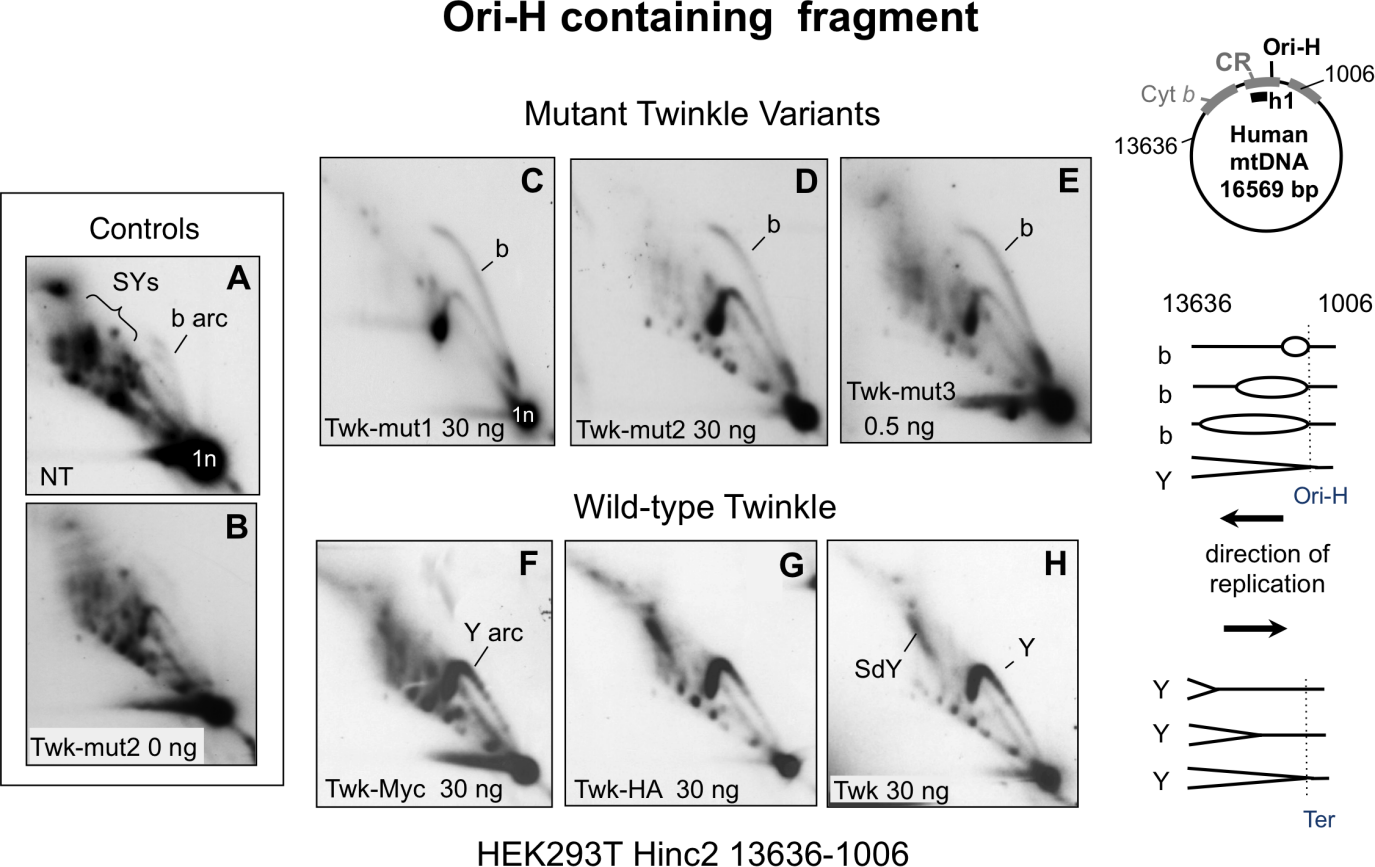
Elevated expression of wild-type Twinkle converts supra-Y arcs to standard replication fork arcs without an accompanying initiation arc. DNA of HEK293T cells was digested with Hinc2 and the products separated by 2D-AGE, and blot hybridized to a radiolabeled probe (h1) that detected the fragment of mtDNA spanning nt 13,636-1,006 (panels **A–H**). DNA samples derived from transformant cell lines carrying no transgene (**A**) or untagged Twinkle with no induction (0 ng doxycycline). (**B**); transgenic Twinkle with deleterious point mutations (**C–E**) (see Supplementary Figure S1), or without (**F–H**); with a C-terminal myc (**F**) or HA (**C–E** and **G**) tag; or untagged (**H**). Expression of the transgenes was induced with the indicated dose of doxycycline for 72 h, and confirmed by quantitative RT-PCR and immunoblotting (Supplementary Figure S7). Y – standard replication fork arc; SY - spra-Y arc; SdY – supra-double Y arc (see text, Supplementary Figures S2 and S5 and (10,14)); b – bubble or initiation arc; 1n – unit length fragment; i.e. 3.9 kb for the Hinc2 fragment nt 13,636-1,006. The Y and b arcs are designated fully duplex DNA intermediates on the basis of their 2D gel mobility (above), and enzyme resistance (Supplementary Figure S2). To the right interpretations of the b and Y arcs, assuming that in all cases a θ mechanism of replication is operating (see also Figure 6).

Transgenic expression of a range of tagged wild-type and mutant variants of Twinkle in human cells (see Materials and Methods), increased the abundance of replication intermediates (RIs) comprising duplex DNA on all branches (class 1 intermediates) that resolve on conventional replication fork (Y) arcs after 2D-AGE (Figures 1 and 2). There was a concomitant decrease in the supra-Y arcs (SYs) comprising (class 2) intermediates of the strand-asynchronous mechanism of mtDNA replication, which in contrast to the class I intermediates are sensitive to RNase H and single-stranded nuclease (Supplementary Figure S2).

Although both transgenic wild-type and mutant Twinkle variants markedly increased the abundance of class 1 mtRIs compared to controls (Figure 2 and Supplementary Figure S2), wild-type Twinkle (with or without a tag) produced a unique pattern: the fragment containing the control region (CR), which includes the major start site(s) for leading strand DNA synthesis (Ori-H), lacked an initiation or ‘bubble’ arc (Figure 2F–H). The absence of a bubble arc suggests there is no initiation of replication in the CR (which in humans spans nucleotide positions (np) 16 024–16 569 and 1–576 (23)), and instead that high levels of functional Twinkle induce replication initiation outside the CR. Therefore, we infer that while defective forms of Twinkle induce replication stalling, the initiation site remains the same as in controls. If true then the nascent strands of mtDNA generated in the presence of mutant Twinkle would be similar to control cells, whereas they might differ in the case of transgenic wild-type Twinkle.

**Figure 3.**
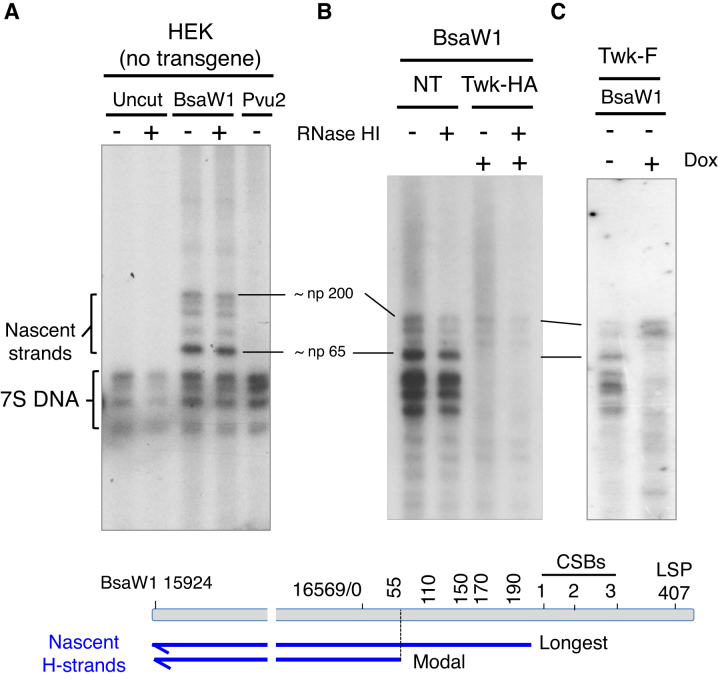
Nascent strands mapping to OriH and 7S DNAs are repressed by elevated expression of wild-type, but not mutant, Twinkle. Nascent strand analysis of sucrose-gradient purified mtDNA from HEK293T cells with no transgene (**A**), or transgenic Twinkle (**B**) and (**C**). Where indicated mtDNAs were left uncut, or digested with BsaW1 or Pvu2; treated with (+) or without (–) *E. coli* RNase HI; and transgene induced (+) with 10 ng doxycycline (Dox) for 72 h. Twk-HA and Twk-F, wild-type Twinkle tagged with HA or FLAG, respectively. Prior to fractionation by 1D-AGE (2% sodium borate) and hybridization with a riboprobe h-H15 869-168 of human H-strand mtDNA, all samples were denatured in 80% formamide, 15 min at 85°C. Markers are shown in Supplementary Figure S3.

Restriction digestion, denaturation and high-resolution 1D-AGE can be used to map ends of DNA to a resolution of 10–20 nucleotides (25). This approach suggests there is a one major Ori-H locus in mouse liver and embryonic fibroblasts (np 16 035) (25), and one in human fibroblasts (24), although several other sites for Ori-H of human mtDNA have also been proposed (27–30). HEK cells have the same prominent nascent strand mapping approximately to np 65 (Ori-H), as human fibroblasts (24), as well as some species of greater length extending to conserved sequence box 1 (CSB1), all of which are resistant to RNase HI (Figure 3A). In HEK cells expressing transgenic wild-type Twinkle the species corresponding to Ori-H were barely detectable, whereas the longer nascent strands were more prominent (Figure 3B, C and Supplementary Figure S3). Taken together with the 2D gel data (Figure 2F–H), these results suggest that the major species at np 65 is the origin of unidirectional replication, Ori-H, that gives rise to the initiation (bubble) arc, and that a surfeit of Twinkle represses initiation of replication at this site, and instead favors initiation outside the CR (Supplementary Figures S3E and F).

**Figure 4.**
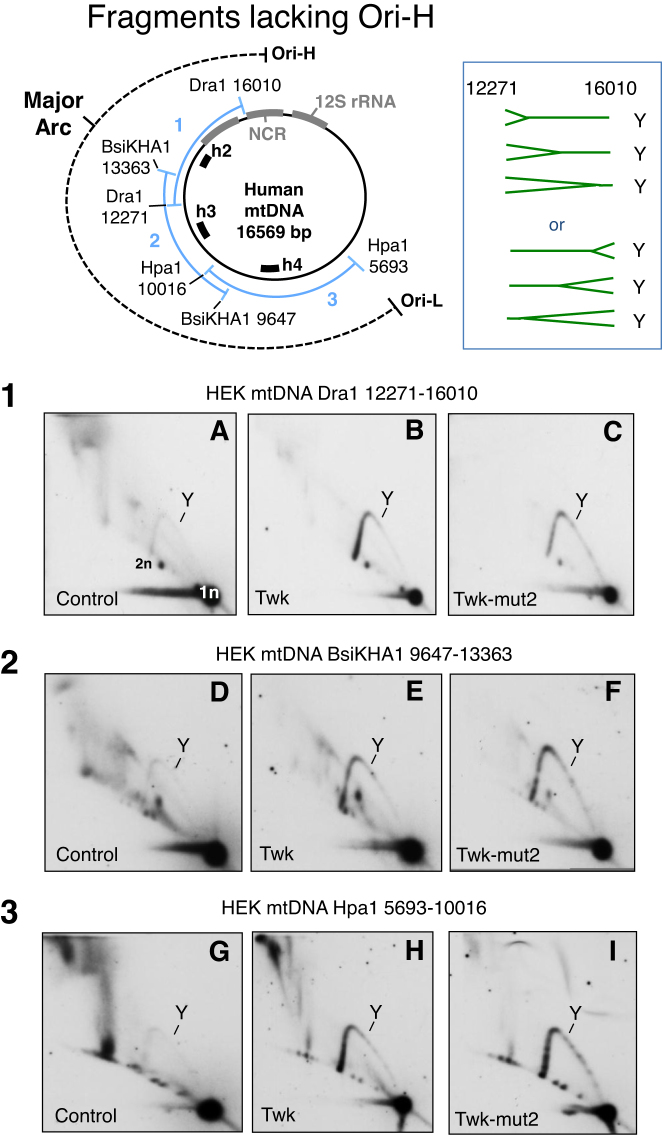
Elevated expression of wild-type or mutant Twinkle increases the abundance of fully-duplex DNA replication intermediates across the major arc of mtDNA. Mitochondrial DNA was analyzed by 2D-AGE from control HEK293T cells, or cells induced to express HA-tagged wild-type or mutant (D311A) Twinkle, after digestion with 1) Dra1, 2) BsaKHA1 or 3) Hpa1. Individual fragments were detected via hybridization to probes h2, h3 or h4 (see schematic map and Supplementary data). Y - replication fork arc, 1n – unit length fragment, 2n - twice the unit length fragment where the Y arc terminates. Standard Y arcs do not distinguish the direction of replication fork movement, and so forks could be entering from either end of all the fragments shown, as illustrated to the right of the map for the Dra1 fragment.

Fragments of mtDNA in the major arc lacking the CR yielded a more prominent standard Y arc, when either wild-type or mutant Twinkle transgenes were expressed in HEK293T cells, but none had a prominent initiation arc (Figure 4). The absence of a major discreet origin of replication suggests high levels of wild-type Twinkle cause replication to initiate at disparate sites, as occurs in nuclear DNA in some contexts (31,32), and previously documented for a minority of replicating mtDNA molecules *in vivo* (12).

### Transgenic Twinkle enhances replication firing across an initiation zone

**Figure 5.**
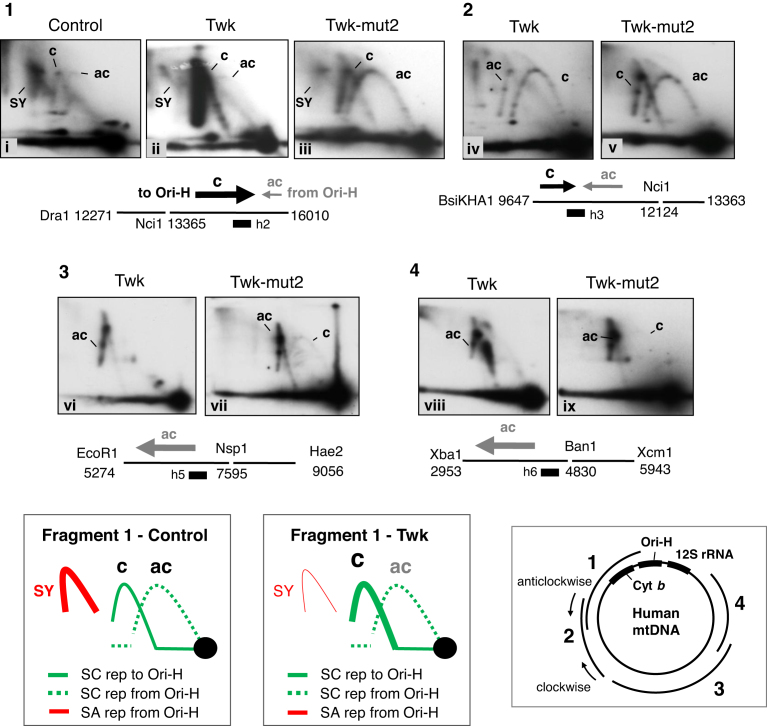
The proportion of replication forks travelling towards, rather than away from, the unidirectional origin Ori-H is markedly increased in cells expressing transgenic wild-type Twinkle. Panels **1–4** Restriction digested HEK293T cell DNA separated by 1D-AGE and then subjected to an additional in-gel digestion step with another restriction enzyme, prior to standard 2D-AGE and gel processing. Fork direction is defined as clockwise (c), or anti-clockwise (ac) as indicated on the schematic map (bottom-right). Assignments of the arcs associated with fragment 1 of control cells (no transgene) and cells expressing wild-type Twinkle (Twk) are shown below the gel images; SC – strand coupled, SA – strand asynchronous replication. Twk and Twk-mut2 (D311A) transgenes were induced with 3 ng/mL doxycycline for 72 h. Immediately below each gel image (**1–4**) the relevant region of mtDNA is represented as a horizontal line, broken at the in-gel digestion site. Arrows above the line indicate the direction of fork travel, the thickness and length of the arrows correspond approximately to the proportion of forks travelling in a particular direction. Black arrows – forks travelling clockwise (towards Ori-H); gray arrows – forks travelling anti-clockwise (away from Ori-H). Black boxes indicate the position of the probes, h2, h4, h5 and h6.

If replication initiates at multiple sites outside the CR (Supplementary Figure S3F), then the number of replication forks entering a fragment from one particular direction will depend on the breadth of the initiation zone and the position of the fragment in the mitochondrial genome, whereas for unidirectional replication from Ori-H, all forks travel in the same direction in every fragment (‘anticlockwise’ — as illustrated on the map of mtDNA, Figure 5). Therefore, as a further test of the idea that transgenic Twinkle promotes initiation of replication outside the CR we determined the direction of travel of the replication forks via fork-direction gel analysis (11,22). First we analyzed a fragment of mtDNA adjacent to the CR but lacking Ori-H: a Dra1 fragment spanning np 12 271–16 010, cut additionally in-gel with Nci1 at np 13 363. In the control HEK293T cells, the strongest arc was a supra-Y arc that is indicative of strand-asynchronous replication ((10) and references therein). In addition, two standard Y arcs (duplex DNA on all branches) were detected, demonstrating that replication forks travel toward, as well as away from, Ori-H in the CR (Figure 5.1-i, and further explained and interpreted in Supplementary Figure S4). The arc corresponding to forks traveling ‘clockwise’ towards the CR (c) was stronger than the ‘anti-clockwise’ (ac) arc (Figure 5.1-i). The former is incompatible with replication initiating in the CR of mammalian mtDNA, i.e. unidirectional replication from Ori-H, but fully concordant with replication initiating from the zone (Ori-z) defined previously (12). In the cells expressing wild-type Twinkle the c-arc was much more pronounced than in control cells, while the supra-Y arc was greatly diminished and the ac-arc remained barely visible (Figure 5.1-ii). Thus, expression of transgenic wild-type Twinkle was accompanied by a marked increase in replication forks advancing towards Ori-H; i.e. most initiation events occur across Ori-z, and few start at Ori-H or any other position in the CR, as inferred from the standard 2D gel analysis (Figure 2F–H) and nascent strand analysis (Figure 3B, Supplementary Figure S3B). Elevated expression of Twinkle also alters the labeling pattern of replicating single molecules of mtDNA and is compatible with DNA synthesis initiating at sites other than Ori-H and Ori-L (Supplementary Figure S4 of (33)).

In the case of an initiation zone adjacent to the CR, fragments of mtDNA mapping progressively further from Ori-H will contain fewer and fewer forks travelling clockwise towards Ori-H. Concordant with this prediction, moving further round the major arc, and away from the CR, the ac-arc became progressively stronger and the c-arc correspondingly weaker in cells expressing transgenic Twinkle (Figure 5.2–4).

### Strand-asynchronous and strand-coupled DNA synthesis are both θ mechanisms of replication

**Figure 6.**
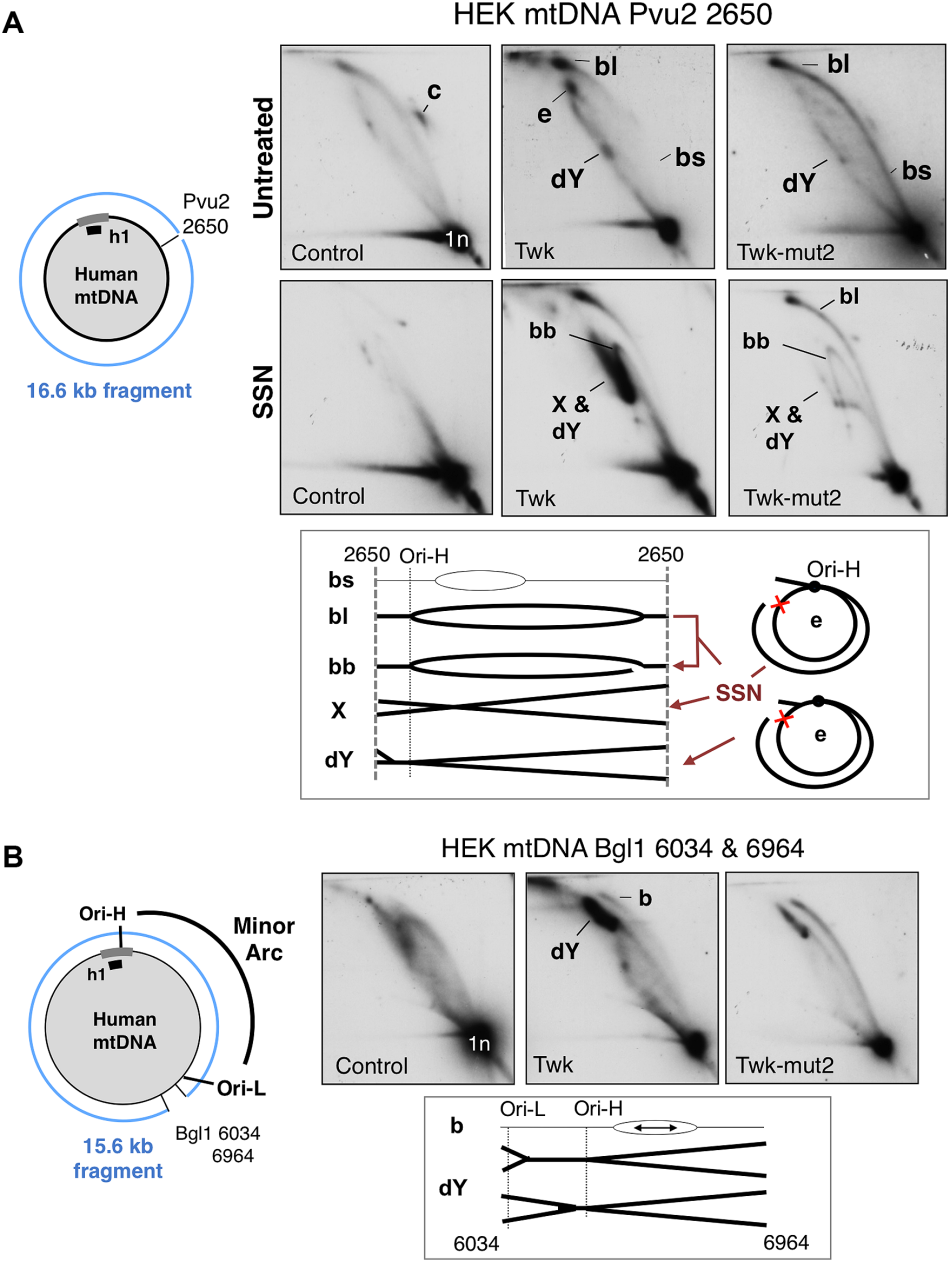
Elevated Twinkle expression induces biphasic θ replication in mtDNA. 2D-AGE analysis of HEK293T cell DNA from cells expressing no transgene (control); transgenic wild-type Twinkle (Twk) or Twinkle D311A (Twk-mut2), both with HA tags. Whole cell DNA was digested with (**A**) Pvu2 that cuts mtDNA at a single site in the minor arc (nt 2650), or (**B**) Bgl1 (sites at np 6034 and 6964). SSN, single-strand-nuclease. Interpretations of the mtRIs are shown beneath the gel images, b – bubble structures, dY – double Y arc. The strong dY is indicative of slow-replication in the minor arc (as per the map to the left of **B**), mtDNA was detected with probe h1. Interpretations of the mtRIs are shown beneath the gel images, c – circles, b – bubble structures (l – large, s – small), dY – double Y arc, e – eyebrow (the red cross indicates a blocked site, which is attributable to RNA/DNA hybrid as the dY arc associated with Bgl1 digests that encompasses the minor arc is SSN-sensitive (Supplementary Figure S5A)). Some loss of RNA/DNA hybrid is expected during extraction because the source material was cultured cells and there was no purification of mitochondria, both of which result in RNA loss and the appearance of gaps that enable SSN to convert b and e forms to broken bubbles (bb), dY and X structures (termination intermediates), as illustrated beneath the gel images of panel A (see text and (14,51) for further details).

Bubble arcs are diagnostic for θ replication (21), but bidirectional θ replication initiating across a broad initiation zone will only give rise to a pronounced bubble arc if the fragment analyzed is larger than the initiation zone. Conveniently, animal mitochondrial genomes are small enough to resolve full-length molecules by 2D-AGE (14). Analysis of linearized full-length mtDNAs revealed a bubble arc extending throughout much of the length of the molecule in control cells *and those expressing transgenic mutant or wild-type Twinkle* (Figure 6A). Hence, the cells expressing transgenic wild-type Twinkle continue to operate a θ mode of replication, as opposed to rolling circle replication (34), or recombination dependent replication (35).

### Strand-coupled DNA synthesis induced by elevated Twinkle expression extends only two-thirds of the way around the mtDNA molecule

Mutant Twinkle enhanced all parts of the initiation arc (Figure 6A) consistent with frequent replication stalling (18). In contrast, wild-type Twinkle produced a biphasic initiation arc; small bubbles were barely detectable, whereas large bubbles were more prominent than normal (Figure 6), indicating the rate of fork progression (DNA synthesis) differs markedly in different regions of the mtDNA: initially replication is so rapid the bubble arc is at the limit of detection, whereas towards the end of the replication cycle it is not only slower than in controls, but slower than in cells expressing mutant Twinkle that suffer replication stalling (Figure 6A). The replication slow zone was more clearly defined when the Pvu2 digested samples were treated with single-stranded nuclease (SSN), resulting in broken bubbles that formed a strong double Y arc preceded by a similarly strong descending Y arc (Figure 6A). To a first approximation molecules in these regions of the 2D gels correspond to the RIs across the minor arc of mtDNA, and this inference was corroborated by digesting the human mtDNA samples with Bgl1 whose site at np 6,034, close to Ori-L (np 5,575), creates a double Y arc encompassing the minor arc of human mtDNA. This double Y arc was greatly enhanced in cells expressing transgenic Twinkle (Figure 6B), indicating a marked inhibition of replication in the final third of the molecule; i.e. between Ori-L and the replication terminus (Ori-H). Elevated Twinkle expression in Drosophila also produced region specific changes in replication, accelerating the process in some areas, by alleviating pausing, while causing fork stalling at others (36). In human cells, impeded replication coincided with a reinstatement of strand-asynchronous (bootlace) replication, as evidenced by the modification of the mtRIs of the minor arc by SSN (Figure 6A) and RNase HI (Supplementary Figure S5A), the multiple blocked sites of the supra-double Y arc (Figure 2G, H and Supplementary Figure S5B) (which was also RNase HI and SSN sensitive (Supplementary Figure S2)), and the strong strand-asynchronous replication fork arc in a fragment spanning np 2650–6324 (Supplementary Figure S5C).

### Elevated Twinkle expression decreases mitochondrial DNA and transcript levels

**Figure 7.**
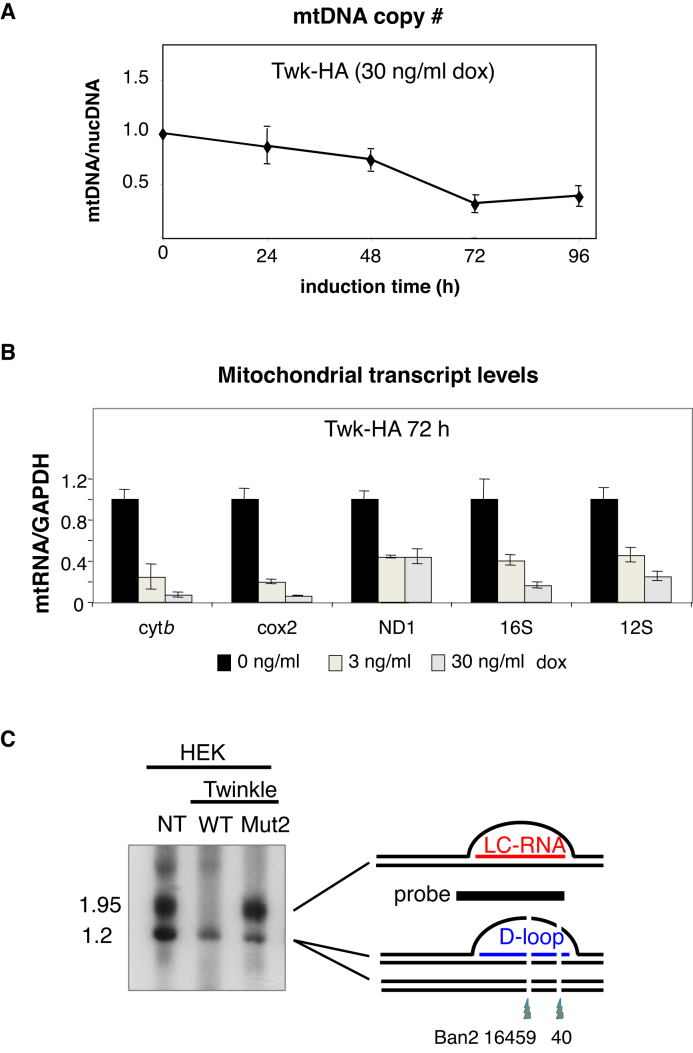
Transgenic wild-type Twinkle causes mtDNA depletion and depresses transcript levels. Whole cellular DNA and RNA were isolated from control HEK293T cells or cells expressing Twinkle-HA or a mutated Twinkle-HA (D311A). (**A**) Relative mtDNA copy number was calculated from the abundance of the cytochrome *b* gene of mtDNA relative to the single copy nuclear gene APP1, using real-time PCR quantification. (**B**) The abundance of five mitochondrial transcripts, cytochrome *b*, cytochrome c oxidase II, NADH dehydrogenase 1, 16S and 12S rRNA determined by RT-q-PCR. (**C**) Ban2 and Acc1 digested whole cell DNAs of HEK cells, without a transgene (NT) or expressing transgenic Twinkle wild-type (WT) or D311A (mut2), were subjected to 1% agarose, 1D-AGE and Southern hybridization to a probe spanning np 15 869-168 of human mtDNA. Two restriction sites are blocked where an R-loop of LC-RNA is present, as illustrated to the right of the gel image (details in (24)).

Mutant forms of Twinkle studied previously caused mtDNA depletion when expressed in HEK293T cells (18), and this was true of the Twinkle D311A mutant (mut2) studied here (Supplementary Figure S6A). Induction of HA tagged wild-type Twinkle for 72 h also resulted in a decrease in mtDNA copy number, to 20% of that of cells maintained without doxycycline (Figure 7A), which can be attributed to the marked inhibition of mtDNA replication in the minor arc region, detailed above.

According to the bootlace model, strand-asynchronous replication depends on transcript incorporation (10); if transcripts are important to the mechanism then their scarcity should restrict or alter mtDNA replication. This raised the question: are many of the effects of elevated Twinkle expression on mtDNA replication (Figures 2–6) a result of changes in transcript levels? Therefore, we analyzed the effect of transgenic Twinkle on mitochondrial RNA (mtRNA) levels. Induction of wild-type Twinkle decreased the steady-state levels of all five mitochondrial transcripts screened compared to mtRNA from the same cells cultured in the absence of doxycycline (Figure 7B). The higher dose produced a mean decrease in mitochondrial RNAs of 82%, in the case of wild-type Twinkle, whereas transcript to mtDNA ratios were higher than normal in cells expressing transgenic mutant Twinkle (Supplementary Figure S6). Thus, the increase in bidirectional strand-coupled mtDNA replication was associated with reduced levels of mitochondrial transcripts; and with a marked decrease in the R-loops in the CR (Figure 7C) (24).

The correlation between transcript levels and the mechanism of replication extended to distinct regions of the mtDNA within a sample, as mtRNA depletion was not uniform across the mitochondrial genome. The three transcripts mapping to the minor arc of mtDNA (ND1 mRNA, 12S and 16S rRNA) were less affected by transgenic Twinkle than the two in the major arc (Cox2, Cyt *b*) (Figure 6B). From the mtDNA copy number data (Figure 7A) we calculated the relative ratio of transcript to mtDNA compared to untreated cells: the three minor arc transcripts were 85% (16S rRNA), 130% (12S rRNA) and 220% (ND1 mRNA) of normal, whereas cytochrome c oxidase II and cytochrome b mRNAs were 35% and 28% of normal, respectively. Thus, while heightened Twinkle expression depressed transcript levels of mRNAs (and the CR R-loops) mapping to the major arc, there was little or no shortage of transcripts in the minor arc of mtDNA, thereby establishing a clear correlation between the abundance of transcripts and the mechanism of mtDNA replication. That is, strand-coupled DNA synthesis was dominant across the major arc where transcripts were scarce, whereas strand-asynchronous (bootlace) replication continued to operate in the presence of high levels of Twinkle in the minor arc where transcripts were present at their usual abundance, per molecule of mtDNA.

## DISCUSSION

According to the bootlace mechanism, processed transcripts are successively hybridized to the lagging template strand during strand-asynchronous mtDNA replication (10,16). In contrast, the original version of the strand-asynchronous mechanism did not envisage any role for transcripts, rather the displaced lagging strand template was said to be coated with mtSSB during the prolonged delay between the initiation of first and second strand DNA synthesis (9). Here, we show that there is little or no strand-asynchronous mtDNA replication when and where there is a dearth of transcripts, as predicted by the bootlace mechanism of mtDNA replication. Hence, we conclude that strand-asynchronous mitochondrial DNA replication is substantially a transcript-dependent mechanism.

### Clarification of the two mechanisms of mtDNA replication

**Figure 8.**
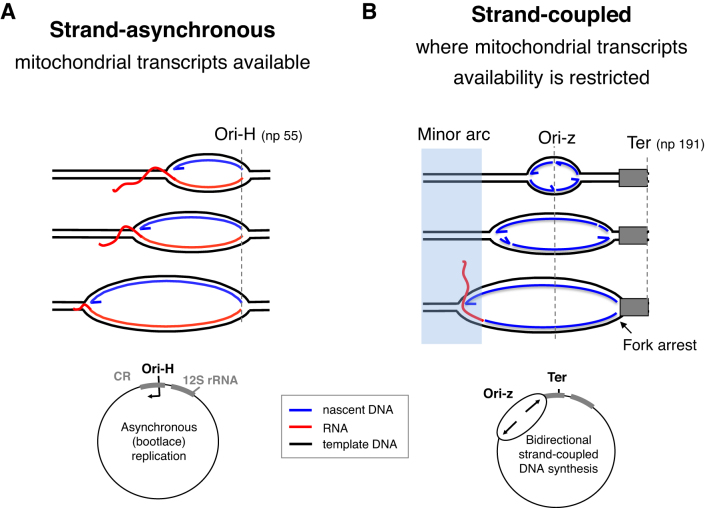
Strand-asynchronous mtDNA replication is a transcript-dependent mechanism. The usually predominant strand-asynchronous mechanism of mtDNA replication initiating at Ori-H (**A**), can be repressed and replaced by one that is bidirectional and initiates across a broad zone (Ori-z) (**B**) (Figures 2–6). Because the relative amount of **A** and **B** correlates with the level of mitochondrial transcripts (Figures 2,7), we infer that transcripts are essential for (or impose) the strand-asynchronous mechanism, and that when transcripts are scarce replication reverts to the ancestral type that is synonymous with that found in the nucleus and in prokaryotes. Although replication stalling also produces replication intermediates that are duplex DNA on all branches (e.g. Figures 2 and Supplementary Figure S2), this does not involve any change in the origin of replication (Figures 2C–E and Supplementary Figure S3B), and is instead attributable to early maturation of the lagging strand RNAs (as illustrated in Supplementary Figure S8).

The types of replication intermediate accompanying high levels of Twinkle documented here are seen in control cells and tissues (11–14,30); they reflect a shift from unidirectional replication to bidirectional replication. The stark contrast achieved clarifies and disentangles features of both mechanisms. It is strand-asynchronous replication that initiates in the CR and is dependent on mitochondrial transcripts, whereas bidirectional, coupled leading and lagging strand DNA synthesis arising from Ori-z is transcript-independent (Figure 8).

A long-standing problem arising from the identification of two classes of replication intermediate in mitochondria (13,14,30) was one of discrimination or compartmentalization. How might mitochondria select one mtDNA molecule for strand-asynchronous replication and another for coupled leading and lagging strand DNA synthesis? Given that strand-coupled DNA synthesis and RNA incorporation on the lagging strand can occur on the same molecule (Figure 6 and Supplementary Figure S5), there is evidently no requirement for a physical demarcation of the two types of replication, and coupled leading and lagging strand DNA synthesis can be inferred to be the default mechanism of replication whenever transcripts are unavailable; in which case any factor that restricts replicating mtDNAs access to RNA will induce strand-coupled DNA replication.

The plasticity of mtDNA replication described here is mirrored in the wider field of DNA replication. ColE1 operates three mechanisms of replication (37); and multiple origins and initiation zones are widespread phenomena (32,38,39), which display redundancy (39,40). Initiation zones can moreover narrow or broaden according to the developmental stage of an organism (41). Hamlin and colleagues demonstrated that none of the 55 kilobase initiation zone of the DHFR locus was essential for the initiation of DNA replication (40), and concluded either that multiple elements were present throughout the region, or else the essential elements lay elsewhere. Similarly, partial deletions of human mtDNA covering all of Ori-z outside the CR, have been catalogued (42), and so it appears the CR contains the critical elements for mtDNA replication, irrespective of the replication start site. A straightforward explanation would be that one or more components of the replication initiation machinery (e.g. Twinkle) is invariably loaded in the CR; however, in the absence of RNA/DNA hybrids, it can translocate to sites across Ori-z before initiating replication. Put another way, RNA/DNA formation in the CR imposes unidirectional replication.

A key feature of mtDNA replication resolved by the new observations is the site of Ori-H, which has been variously been assigned locations at nucleotide positions 54/57, 111, 150, 168 and 191, by 5′ end mapping (27,28,30,43). Here the nascent strands forming a band close to np 65 (and thus inferred to be synonymous with the 5′ends of DNA mapped at the single nucleotide level to np 54/57 (28,30)) disappeared together with the initiation arc when wild-type Twinkle was expressed at a high level, and so they define the start site of leading (heavy strand) DNA synthesis; i.e. Ori-H. In contrast the species mapping to approximately np 200 and a little below (inferred to be nps 150, 169 and 190 identified previously (27–30)) were enhanced by wild-type Twinkle expression, and thus are demonstrably independent of the (Ori-H) initiation arc. Therefore, we propose that the 5′ends mapping to np 150, 169 and 190 mark termini rather than start sites; and consequently, Ori-H in human cells (including primary fibroblasts (24)) is a single site located in the predicted stem-loop spanning np 39–71 of human mtDNA (44).

### Twinkle and the priming of strand-coupled mtDNA replication

Twinkle is a DNA helicase; hence, the straightforward explanation for the switch in replication mechanism reported here is that excess Twinkle unwinds mitochondrial RNAs hybridized to DNA. That transgenic Twinkle interacts directly with RNA/DNA hybrids is implied by the fact that the reduced numbers of mRNAs will still greatly outnumber the mtDNAs; and is evidenced by the near complete loss of the (R-loop) LC-RNA (Figure 7C), which constitutes the first bootlace (24). While the high level of expression achieved in cells is not physiological (Supplementary Figure S7), much lower levels of Twinkle decrease transcript levels in mice (6); hence, a local high concentration of Twinkle sufficient to induce strand-coupled mtDNA replication in vivo may be readily achievable, or it might require coordination with another factor that prevents, or removes, transcripts hybridizing with replicating mtDNA.

Although Twinkle is homologous to the gp4 primase-helicase of the T7 phage (45), metazoan Twinkle appears to lack the ability of its ancient bacteriophage ancestor to synthesize RNA primers (46). However, the increases in strand-coupled replication intermediates induced by transgenic Twinkle suggest it, directly or indirectly, stimulates priming of DNA synthesis on the lagging strand. Thus, human Twinkle may be capable of acting as a primase *in situ*, despite the sequence changes that suggest otherwise (46). Alternatively, Twinkle's ability to anneal two strands of DNA (47), which it shares with its nuclear counterpart (48), could extend to priming via the annealing of oligoribonucleotides (created by the degradation of mitochondrial transcripts) to DNA, in the manner of its bacteriophage homolog (45,49). Equally, Twinkle's annealing capacity (47) could facilitate the hybridization of intact mitochondrial transcripts to the template lagging-strand (and which could also form the basis of an RNA-mediated repair mechanism, as has been proposed for nuclear DNA (50)). Hence, mitochondrial transcripts could regulate both mechanisms of mtDNA replication: when intact being used to impose the bootlace mechanism; and when processed to oligonucleotides, promoting frequent priming on the lagging-strand, both potentially mediated by Twinkle.

## Supplementary Material

Supplementary Data
