# Supplementary material for: Transcript availability dictates the balance between strand-asynchronous and strand-coupled mitochondrial DNA replication
Source: Nucleic Acids Res. 2018 Sep 20;46(20):10771–81. doi: 10.1093/nar/gky852 (PMC6237803; doi:10.1093/nar/gky852)
Supplement: Supplementary Data [file gky852_supplemental_files.pdf]

Supplementary Information

Transcript availability dictates the balance between strand-asynchronous and strand-coupled mitochondrial DNA replication

Supplementary Figures

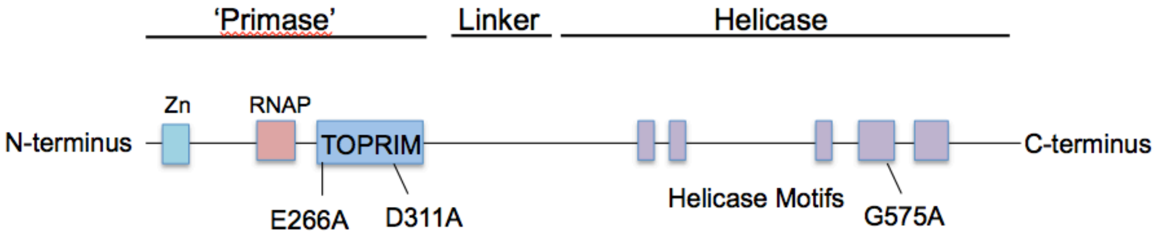

Figure S1. Linear map of Twinkle showing the position of the three mutants analyzed in this study. The enzyme is homologous to the T-odd phage primase-helicase, but the mammalian version of Twinkle appears to lack primase activity. Although there is a distinct helicase domain, mutations in the N-terminal (´primase´ domain) have been shown to be involved in single-stranded DNA binding and to impair helicase function (1).

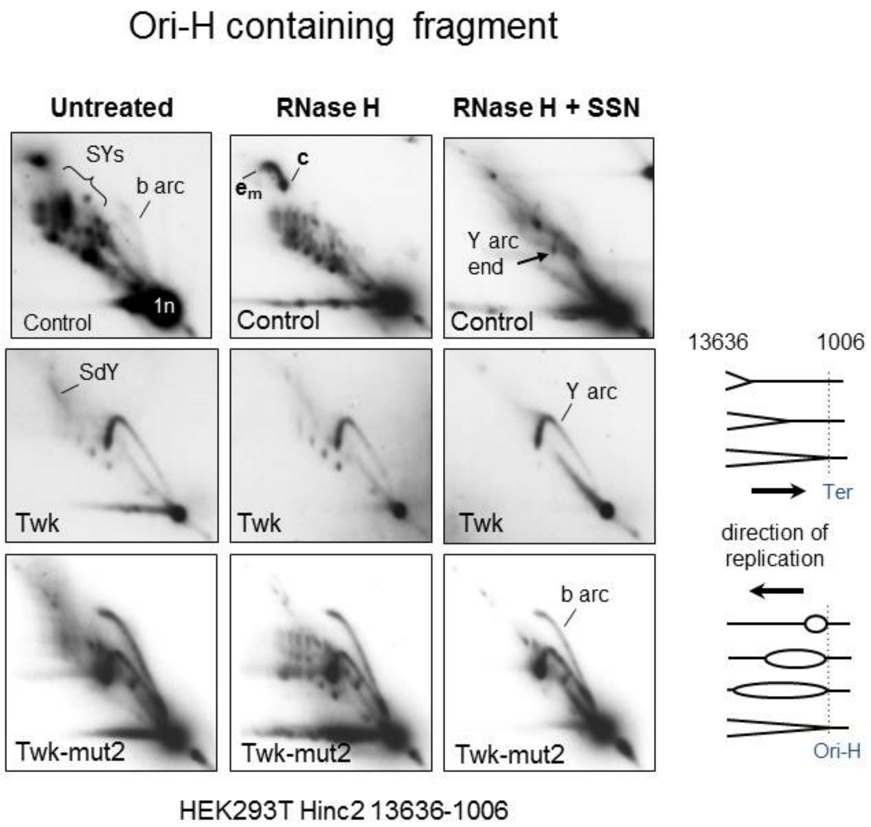

Figure S2. Transgenic Twinkle DNA helicase expressed in HEK293T cells remodels mtDNA replication to render the intermediates resistant to RNase H1 and single-stranded nuclease. 30 ng/mL doxycycline was added to control HEK293T cells and cells carrying Twinkle-HA (mutant or wild-type) transgenes: total cellular DNA was harvested 72 hours later. DNA was digested with Hinc2 and treated additionally with RNase HI and single-stranded nuclease (SSN) as indicated. The products of the reactions were separated by 2D-AGE, transferred to solid support and hybridized to a radiolabeled probe (h1) that detected the fragment spanning nt 13,636-1,006. Y - replication fork arc, SdY - supra-double Y arc; b – bubble or initiation arc, e – eyebrow, e_m- modified eyebrow. Note that transgenic Twinkle expression is associated with Y and bubble arcs that are RNase H and SSN nuclease resistant, which indicates the RIs comprise duplex DNA on all branches. In contrast, the SdY arc is sensitive to these enzymes.

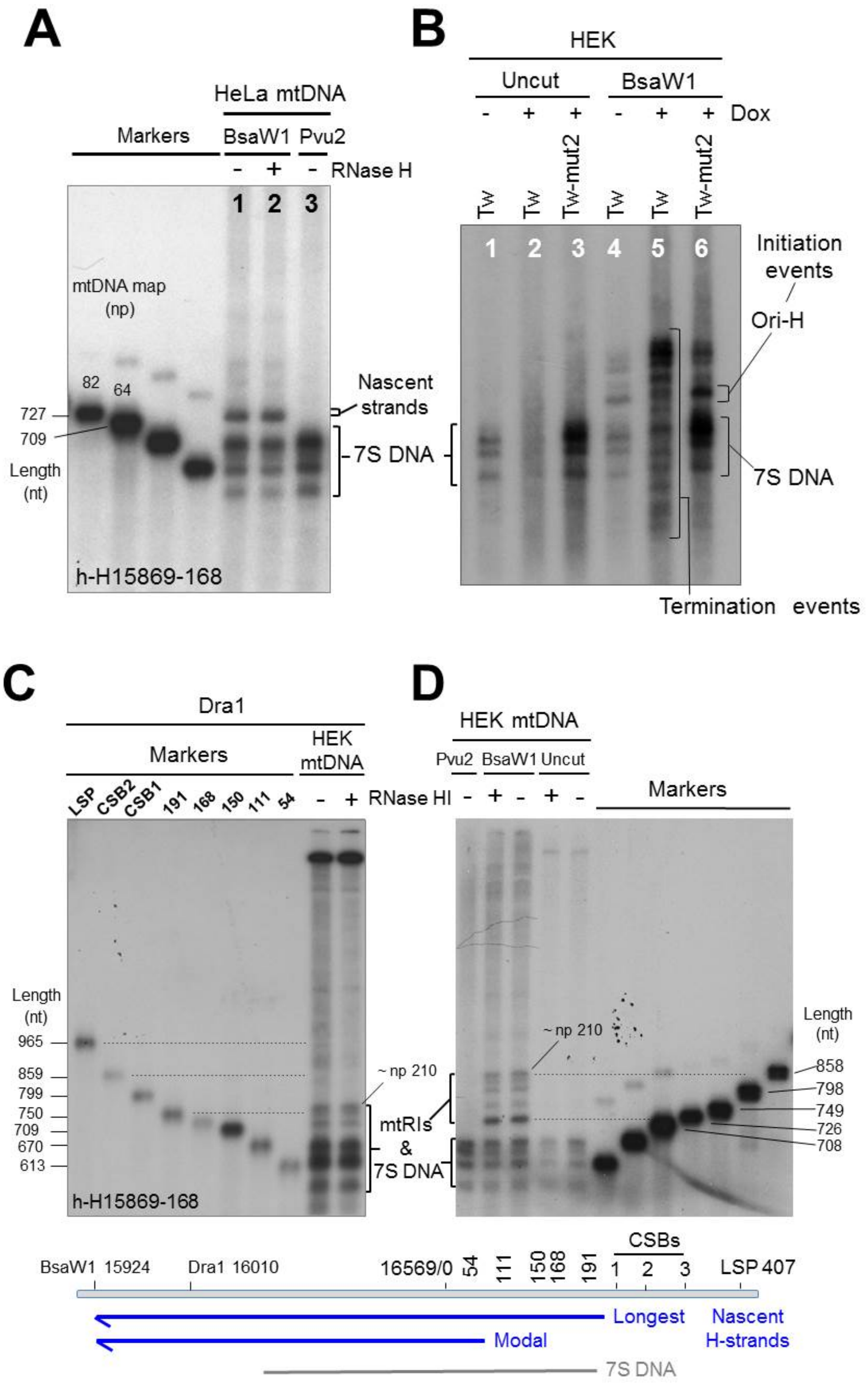
A
HeLa mtDNA
Markers
BsaW1
Pvu2
RNase H
mtDNA map (np)
82
64
727
709
Length (nt)
Nascent strands
7S DNA
h-H15869-168
B
HEK
Uncut
BsaW1
Dox
Tw
Tw-mut2
Initiation events
Ori-H
7S DNA
Termination events
C
Dra1
Markers
HEK mtDNA
LSP
CSB2
CSB1
191
168
150
111
54
RNase HI
Length (nt)
965
859
799
750
709
670
613
~ np 210
mtRIs & 7S DNA
h-H15869-168
D
HEK mtDNA
Pvu2
BsaW1
Uncut
Markers
~ np 210
Length (nt)
858
798
749
726
708
BsaW1 15924
Dra1 16010
16569/0
54
111
150
168
191
CSBs
1
2
3
LSP 407
Longest
Modal
Nascent H-strands
7S DNA

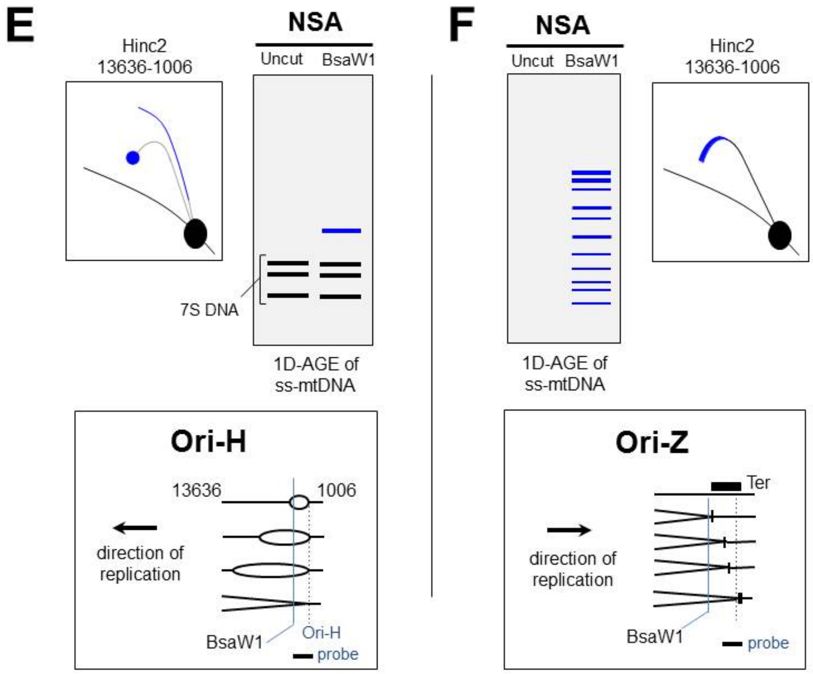

Figure S3. Nascent strands mapping to OriH and 7S DNAs are repressed by elevated expression of wild-type, but not mutant, Twinkle DNA helicase. Sucrose-gradient purified mtDNA from (**A**) HeLa or (**B**) HEK cells digested with BsaW1, fractionated by 1D-AGE after heat and formamide denaturation, and hybridized with a riboprobe spanning nt 15,869-168 of human H-strand mtDNA. The mtDNA was run in parallel with a series of markers, not all of which are shown to reduce clutter, (see (Supplementary) Methods), and from which the lengths of the H-strands and their map positions were estimated. (**A**). After restriction digestion samples were treated additionally with *E. coli* derived RNase HI (+), or not (-). (**B**) - no doxycycline added; + 10 ng doxycycline added 72 h prior to DNA isolation. To note: the pattern of H-strands of DNA is similar in HeLa cells (panel **A**) and control HEK cells (Fig. 3A), in HEK cells carrying transgenic wild-type Twinkle prior to induction of the transgene (panel **B**, lanes 1 and 4); and in HEK cells carrying mutant (D311A) Twinkle after induction (panel **B**, lanes 3 and 6). In contrast, induction of wild-type Twinkle diminishes the band designated Ori-H, together with the 7S DNAs (panel **B**, lanes 2 and 5, and Fig. 3B). Panels (**C**) and (**D**) demonstrate that cutting closer to Ori-H (with Dra1) results in overlap between nascent H-strands of productive replication intermediates and 7S DNAs. Panel **E** illustrates the expected 2D gel pattern for unidirectional replication from a site (Ori-H) in the control region (where all strands are DNA), and which conforms to those observed when mutant forms of Twinkle are expressed in human cells (Fig. 2A-2C). A discreet initiation site will also produce nascent strands (NS) of the same length (blue horizontal line) after restriction digestion with an enzyme such as BsaW1 that cuts human mtDNA at np 15,924, close to the origin, as previously demonstrated for human mtDNA of primary fibroblasts (2,3). (**F**) Conversely, the initiation arc is absent when transgenic wild-type Twinkle is expressed in human cells; Fig 2F-2H (illustrated in the 2D gel cartoon), and the strong signal of the descending Y arc predicts a series of BsaW1 cleaved nascent strands, blue horizontal lines in the 1D gel cartoon. At the foot of the figure the inferred directions of replication, which were corroborated by further analysis (see Figs 4 and 5 and main text). NSA – nascent strand analysis, Ori-z – initiation zone, see text for details and (4). 1D-AGE after restriction digestion and denaturation.

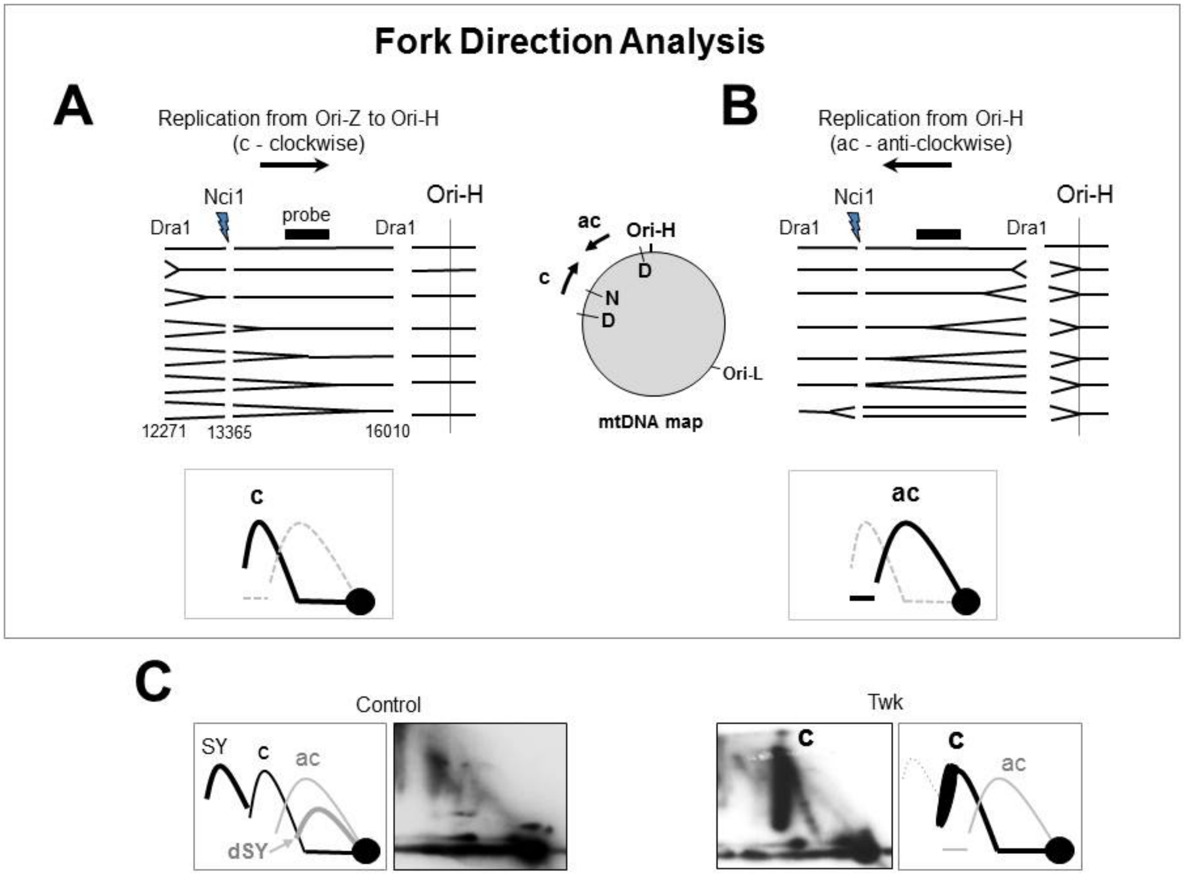

Figure S4. The key predicted and empirical results of the fork direction gel assay. (**A**) and (**B**) Illustrations of replication forks entering the same (Dra1) restriction fragment of mtDNA from opposite ends, and the consequent effects of subsequent cleavage with Nci1 (in-gel) on individual replication intermediates and on Y arc mobility (insets). If forks enter from both ends a composite pattern will be produced (gray broken line). (**C**) Control HEK293T cells yielded arc c that is incompatible with initiation of replication from Ori-H, and this was greatly enhanced by transgenic HA tagged Twinkle (Twk). The supra-Y arc (SY) (and its partially degraded derivative – dSY) results from unidirectional replication from the CR (5). 'Splaying' of the prominent descending portion of the Y is attributable to some branch migration occurring during the in-gel digestion step.

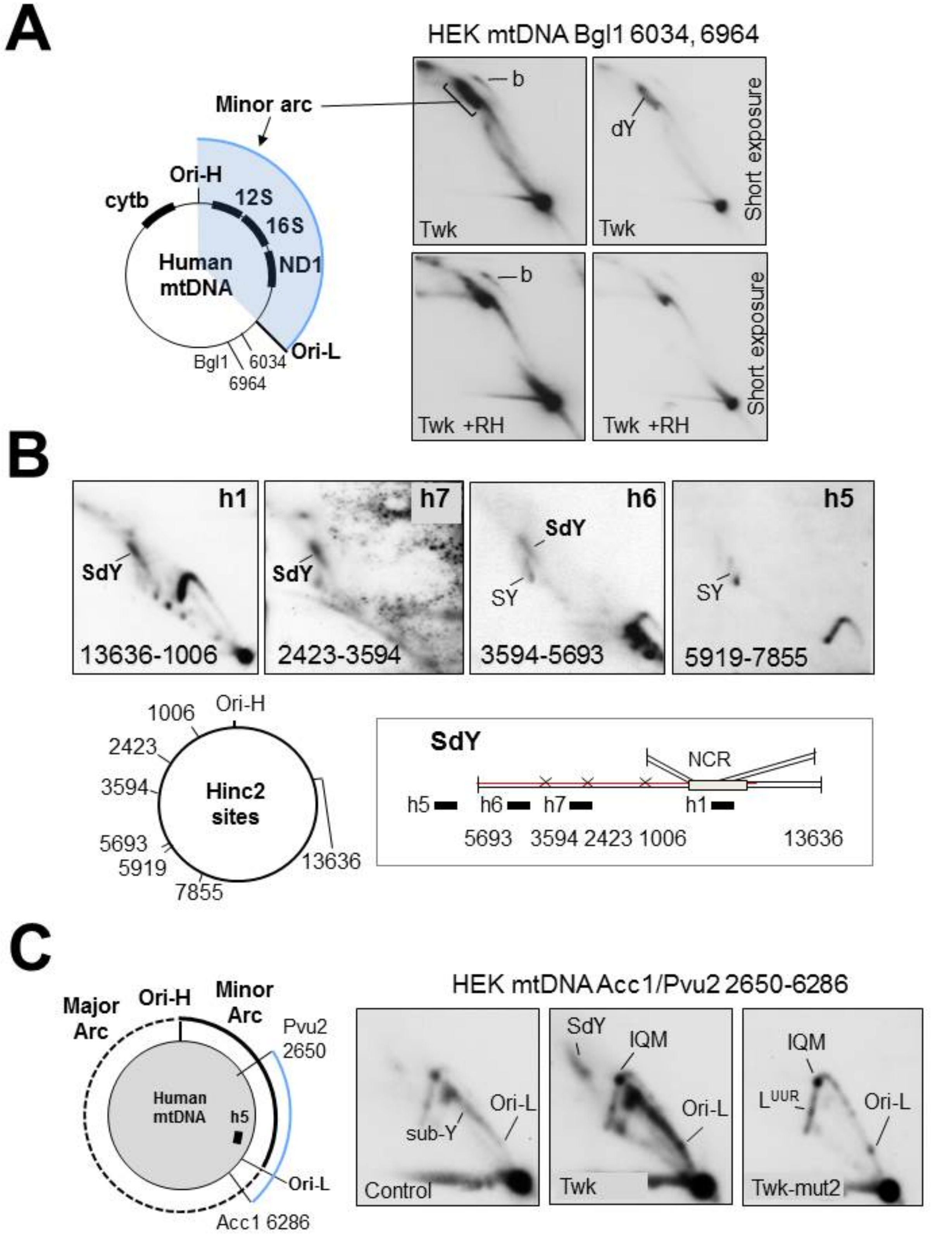
A
HEK mtDNA Bgl1 6034, 6964
Minor arc
Ori-H
cytb
12S
16S
ND1
Human mtDNA
Ori-L
Bgl1
6034
6964
b
dY
Twk
Short exposure
Twk +RH
B
h1
h7
h6
h5
SdY
SY
13636-1006
2423-3594
3594-5693
5919-7855
1006
Ori-H
2423
3594
Hinc2 sites
5693
5919
7855
13636
NCR
5693 3594 2423 1006
C
Major Arc
Minor Arc
Pvu2 2650
Acc1 6286
HEK mtDNA Acc1/Pvu2 2650-6286
sub-Y
IQM
Control
Twk
Twk-mut2

Figure S4

Figure S5. Strand-asynchronous replication is re-established in the minor arc of mtDNA in cells expressing transgenic wild-type Twinkle. (A) The Bgl1-associated prominent double Y-arc (covering the minor arc of human mtDNA), induced by transgenic wild-type Twinkle (Fig. 5B), is modified by RNase H, whereas the bubble arc (corresponding to the major arc of mtDNA) is unaffected by RNase H. **(B)** A novel arc of mtRIs appearing in cells expressing transgenic Twinkle DNA helicase is a double Y species with an extended arm owing to restriction site blockage. 2D blots equivalent to Fig. 1E, 1F were probed to determine the extent of restriction site blockage. The SdY arc is detected by probes h1, h6 and h7, but not h5. The position of the probes and the deduced blocked restriction sites are illustrated on a representative intermediate of the SdY arc (inset). These results indicate that the Hinc2 SdY arc associated with HEK293T cells expressing wild-type Twinkle spans nt 13636-5693, and because it is sensitive to RNase HI and SSN (Fig. S2) it is not formed of duplex DNA on all branches. **(C)** A fragment of human mtDNA (nt 2,650-6,286) spanning much of the minor arc detected by probe h5 yields a strong strand-asynchronous replication arc in samples derived from cells expressing HA tagged Twinkle wild-type (Twk-HA). Loss of tracts of RNA from the lagging-strand branch of mtRIs creates a Y-like arc with a lower trajectory than a conventional Y arc (sub-Y) in fragments spanning the minor arc and including the light-strand ‘origin’ of replication Ori-L (for further details see (6)), b – bubble structures, dY – double Y arc.

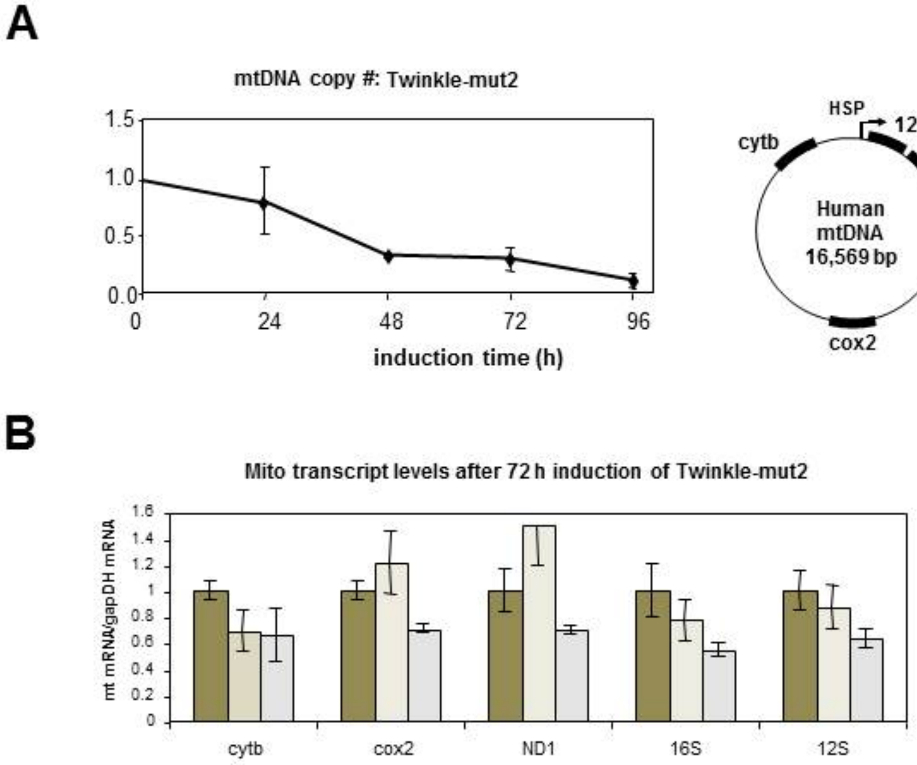

Figure S6. Mutant Twinkle causes mtDNA depletion, accompanied by an increase in transcripts per mtDNA. Whole cellular DNAs were isolated from control HEK293T cells or cells expressing a mutated Twinkle-HA (D311A). Relative mtDNA copy number was calculated from the abundance of the cytochrome *b* gene of mtDNA relative to the single copy nuclear gene APP1, using real-time PCR quantification as previously described (7). (**B**) The abundance of five mitochondrial transcripts, cytochrome *b*, cytochrome c oxidase II, NADH dehydrogenase 1, 16S and 12S rRNA was measured by RT-q-PCR.

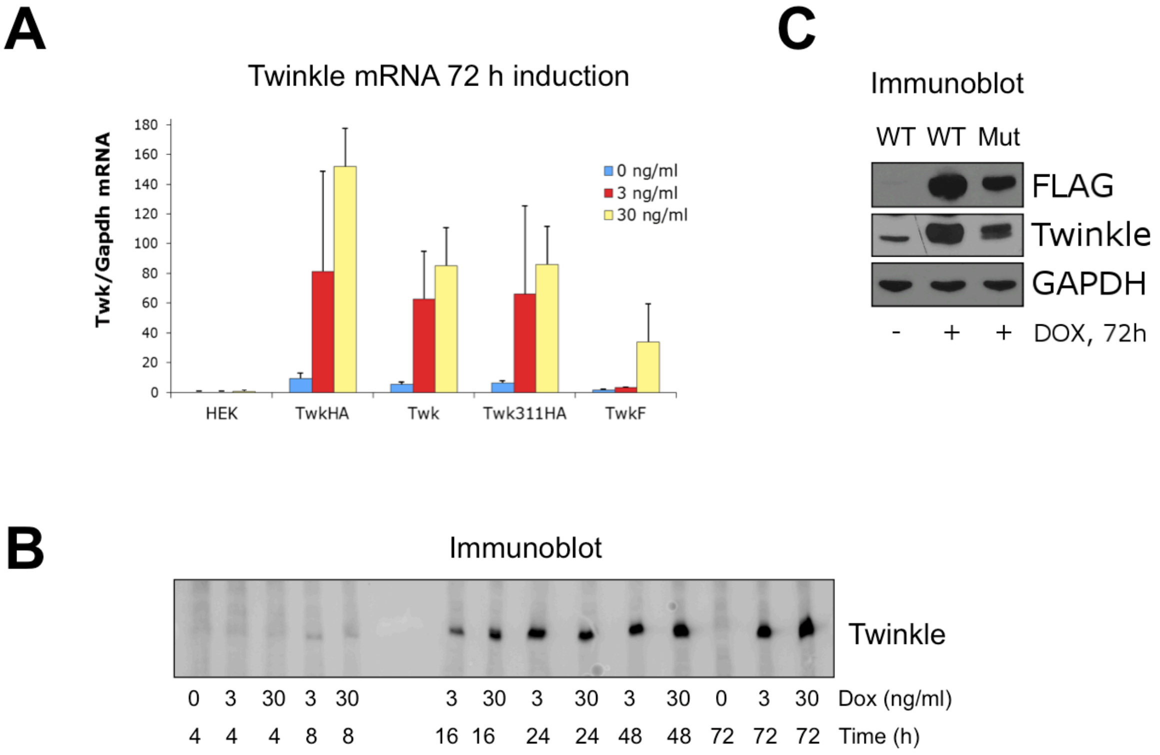

Figure S7. Transgenic Twinkle DNA helicase is inducibly expressed in HEK293T cells. (A) RNA was extracted from HEK293T cells transformed with pcDNA5 plasmids carrying cDNAs to wild-type Twinkle without a tag (Twk), wild-type Twinkle with a haemagglutinin tag (Twk-HA) or a mutant variant of Twinkle D311A (Twk-mut2-HA) and untransformed (control) cells. Twinkle and GAPDH mRNAs levels were determined by Q-RT-PCR and the ratio was expressed relative to control cells (panel **A**, n = 2; panel **B**, n = 3) that was arbitrarily set as 1. The transgene was induced for 24 h or 72 h with 0, 3 or 30 ng/ml doxycycline (Dox). **(B)** Dose and time dependent expression of transgenic Twinkle (wild-type, untagged) via immunodetection. **(C)** Expression of transgenic Twinkle wild-type and mutant (D311A) after 72 h induction with 10 ng/mL doxycycline (FLAG tagged) via immunodetection. This difference in expression was observed in independent transformant cell lines, suggesting HEK cells are less tolerant of the mutant variant, which is further suggested Twk-mut2 causing (slightly) more depletion of mtDNA (Fig. 7A vs S6A). Further increasing the level of transgenic mutant protein would be expected to exacerbate the replication stalling phenotype and accelerate the mtDNA depletion. Moreover, replication stalling was never seen at earlier time points or with lower doses of doxcycline in the cells with transgenic wild-type Twinkle (data not shown). Therefore it is unlikely the difference in effects on mtDNA replication and mtRNA between mutant and wild-type Twinkle (Figs 2-7) are attributable to the amount of transgenic protein. Hence, the data suggest that a considerable excess of ***functional*** Twinkle protein is required to repress strand-asynchronous replication and transcripts, and enhance bidirectional strand-coupled replication in cultured cells.

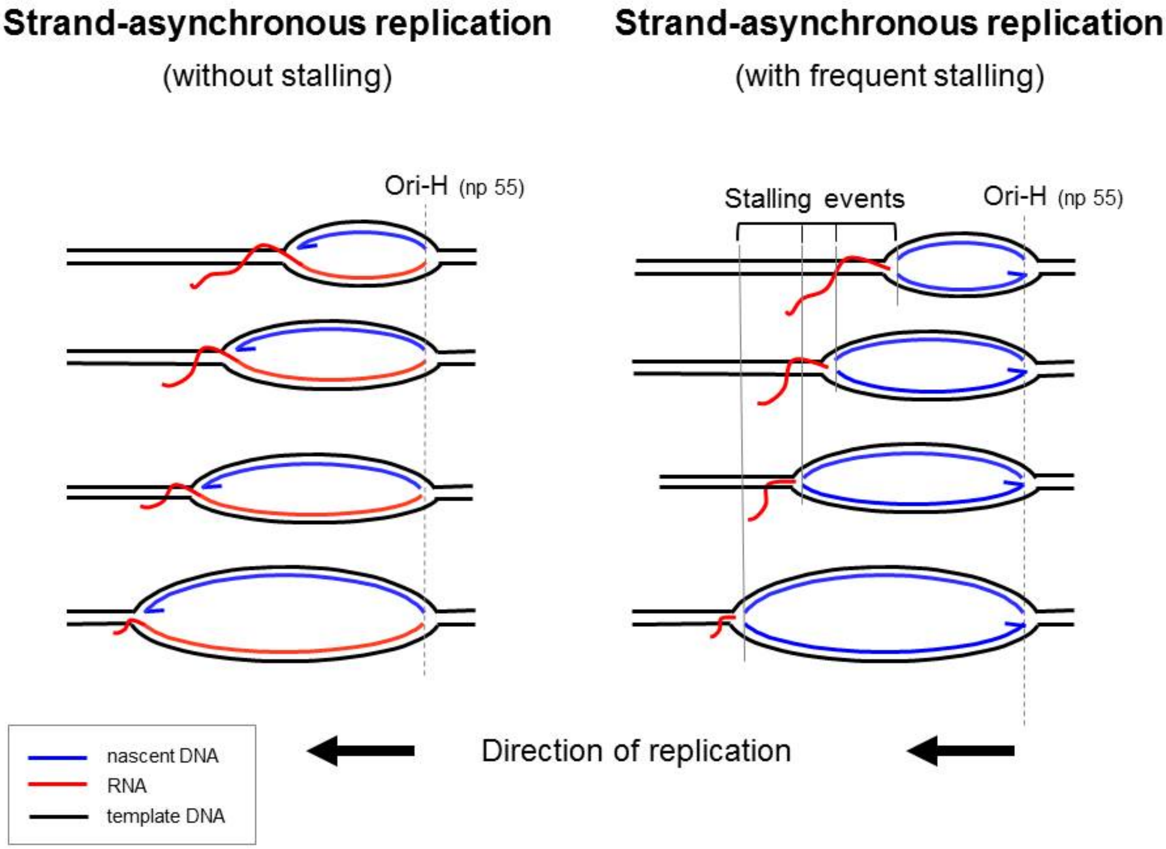

Figure S8. Replication stalling induces early maturation of bootlace RNAs creating replication intermediates that comprise duplex DNA on all branches, without altering the origin of replication. This model is concordant with the data for transgenic expression of mutant Twinkle DNA helicase, which creates replication intermediates that: 1. are duplex DNA on all branches (Supplementary Figure S2); 2. yield a prominent initiation (b) arc in fragments containing Ori-H (Figure 2C-E); and 3. have the same nascent heavy strands of mtDNA as controls (Supplementary Figure S3B). Points 2 and 3 distinguish the cells expressing mutant Twinkle from those expressing wild-type Twinkle. Moreover, the former but not the latter have no shortage of transcripts (per mtDNA) (Supplementary Figure S6) or R-loops (Figure 7C) to prevent the bootlace mechanism operating.

Supplementary Materials and Methods

Primers and probes used for Southern hybridization and RNA quantitation.

**Probe**	**Primer**	**Human mtDNA primer sequences 5´- 3´**	**Region (np)**
h1	F<br>R	CATTACAGTCAAATCCCTTCTCGTCC<br>GAATGAGGCAGGAATCAAAGACAGAT	16,341-151
h2	F<br>R	AACCCCCATAAATAGGAGAAGGCTTAG<br>CGATGTGTAGGAAGAGGCAGATAAAGA	14,587-15,042
h3	F<br>R	AATCAACACAACCACCCACAGCCTA<br>GGCTCAGGAGTTTGATAGTTCT	10,831-11,332
h4	F<br>R	TTATTCCTAGAACCAGGCGACCTGC<br>TTCGTTCATTTTGGTTCTCAGGGTT	7,961-8,535
h5	F<br>R	GCATCACTATACTACTAACAGAC<br>GTGATAAGCTCTTCTATGATAG	6,518-7,051
h6	F<br>R	GTCTCCATACCCATTACAATCTCCAG<br>GTTGCTTGCGTGAGGAAATACTTG	4,220-4,664
h7	F<br>R	ATGACACAGCAAGACGAGAAG<br>GCCAACCTCCTACTCCTCATTG	2,706-3,338
H15869-168	F<br>R	TAATACGACTCACTATAGGAAAATACTCAAATGGGCCT<br>GTCCGGTGCGATAAATAATAGGATGAGG<br>[T7 promoter sequence is underlined.]	15,869-168

**Gene**	**Primer**	**Oligonucleotide sequence 5′ to 3′**
Amyloid precursor protein (APP)	F<br>R<br>P	TTT TTG TGT GCT CTC CCA GGT CT<br>TGG TCA CTG GTT GGT TGG C<br>CCC TGA ACT GCA GAT CAC CAA TGT GGT AG
Cytochrome *b* (cyt *b*)	F<br>R<br>P	GCC TGC CTG ATC CTC CAA AT<br>AAG GTA GCG GAT GAT TCA GCC<br>CAC CAG ACG CCT CAA CCG CCT T
Cytochrome oxidase II	F	CGT CTG AAC TAT CCT GCC CG

(Cox2)	R<br>P	TGG TAA GGG AGG GAT CGT TG<br>CGC CCT CCC ATC CCT ACG CAT C
16S ribosomal RNA	F<br>R<br>P	TTT GCA AGG AGA GCC AAA GC<br>AGA CGG GTG TGC TCT TTT AGC T<br>AGA CCC CCG AAA CCA GAC GAG CTA CC
12S ribosomal RNA	F<br>R<br>P	CCC CAG GTT GGT CAA TTT C<br>CGG CTT CTA TGA CTT GGG TTA A<br>TGC AGC CAC CGC GGT CA
NADH dehydrogenase 1 (ND1)	F<br>R<br>P	CCC TAA AAC CCG CCA CAT CT<br>AGA GCG ATG GTG AGA GCT AAG G<br>ATC ACC CTC TAC ATC ACC GCC CCG
GAPDH	F<br>R<br>P	GAA GGT GAA GGT CGG AGT CAA C<br>CAG AGT TAA AAG CAG CCC TGG T<br>TTT GGT CCG TAT TGG GCG
Twinkle	F<br>R<br>P	GGG CGA GCT GAC GGT CTT<br>GGC ATA CTC ACT GAT GAA TGT CG<br>ACA GGG CCA ACA GGC AGT GGA AAG

Primers for markers of defined length: for high-resolution 1D-AGE (Figures 2 and S2) were generated by PCR amplification of human mtDNA with forward primer:

h-15869 5´-AAAATACTCAAATGGGCCTGTCC-3'´
and the following series of reverse primers:

h-54 5´-CCAAATGCATGGAGAGCTCC- 3';
h-111 5-TGCTCCGGCTCCAGCGTC- 3';
h-150 5´-GATGAGGCAGGAATCAAAGACA- 3';
h-168 5´ -GGTGCGATAAATAATAGGATGAGG- 3';
h-191 5'-TGTTCGCCTGTAATATTGAACG-3´;
h-240 5'-TATTATTATGTCCTACAAGCAT-3';´
h-300 5'-TGGTGGAAATTTTTTGTTATGATG-3';
h-407 5'-AAAGATAAAATTTGAAATCTGGT-3'.

PCR products were digested with Dra1 to create a consistent end at np 16,010 prior to 1D-AGE fractionation of the markers. Another marker spanning the BsaW1 site at np 15,924 to 50 was generated by digesting 143B crude mtDNA with BsaW1 and BstX1 to create a product of 695 bases in length.

Supplementary References

1. Farge, G., Holmlund, T., Khvorostova, J., Rofougaran, R., Hofer, A. and Falkenberg, M. (2008) The N-terminal domain of TWINKLE contributes to single-stranded DNA binding and DNA helicase activities. *Nucleic acids research*, **36**, 393-403.
2. Akman, G., Desai, R., Bailey, L.J., Yasukawa, T., Dalla Rosa, I., Durigon, R., Holmes, J.B., Moss, C.F., Mennuni, M., Houlden, H. *et al.* (2016) Pathological ribonuclease H1 causes R-loop depletion and aberrant DNA segregation in mitochondria. *Proc Natl Acad Sci U S A*, **113**, E4276-4285.
3. Holmes, J.B., Akman, G., Wood, S.R., Sakhuja, K., Cerritelli, S.M., Moss, C., Bowmaker, M.R., Jacobs, H.T., Crouch, R.J. and Holt, I.J. (2015) Primer retention owing to the absence of RNase H1 is catastrophic for mitochondrial DNA replication. *Proc Natl Acad Sci U S A*, **112**, 9334-9339.
4. Bowmaker, M., Yang, M.Y., Yasukawa, T., Reyes, A., Jacobs, H.T., Huberman, J.A. and Holt, I.J. (2003) Mammalian mitochondrial DNA replicates bidirectionally from an initiation zone. *J Biol Chem*, **278**, 50961-50969.
5. Reyes, A., Kazak, L., Wood, S.R., Yasukawa, T., Jacobs, H.T. and Holt, I.J. (2013) Mitochondrial DNA replication proceeds via a 'bootlace' mechanism involving the incorporation of processed transcripts. *Nucleic acids research*, **41**, 5837-5850.
6. Yasukawa, T., Reyes, A., Cluett, T.J., Yang, M.Y., Bowmaker, M., Jacobs, H.T. and Holt, I.J. (2006) Replication of vertebrate mitochondrial DNA entails transient ribonucleotide incorporation throughout the lagging strand. *EMBO J*, **25**, 5358-5371.
7. Tyynismaa, H., Sembongi, H., Bokori-Brown, M., Granycome, C., Ashley, N., Poulton, J., Jalanko, A., Spelbrink, J.N., Holt, I.J. and Suomalainen, A. (2004) Twinkle helicase is essential for mtDNA maintenance and regulates mtDNA copy number. *Hum Mol Genet*, **13**, 3219-3227.
